# Effect of chondroitin sulfate on soluble biomarkers of osteoarthritis: a method to analyze and interpret the results from an open-label trial in unilateral knee osteoarthritis patients

**DOI:** 10.1186/s12891-016-1268-4

**Published:** 2016-10-06

**Authors:** Ingrid Möller, Myriam Gharbi, Helena Martinez Serrano, Marta Herrero Barbero, Josep Verges Milano, Yves Henrotin

**Affiliations:** 1 Poal Institute of Rheumatology, Barcelona, Spain; 2 Artialis SA, GIGA Tower, CHU Sart-Tilman, 4000 Liège, Belgium; 3 Clinical R&D Area, Bioiberica S.A., Barcelona, Spain; 4 Bone and Cartilage Research Unit, Arthropôle Liège, Institute of Pathology, University of Liège, level +5, CHU Sart-Tilman, 4000 Liège, Belgium

**Keywords:** Biomarkers, Pain, Osteoarthritis, Chondroitin sulfate, Cartilage metabolism

## Abstract

**Background:**

The aim of this study was to investigate the effects of chondroitin sulfate (CS) on the serum levels of Coll2-1 in patients with knee OA.

**Methods:**

Seventy two patients with unilateral symptomatic knee OA were involved in a post-authorization open-label study evaluating CS (800 mg/day). The primary outcome was the % relative change in serum Coll2-1 (sColl2-1). The secondary outcomes were the evaluation of pain (VAS) and function (Lequesne’s Index). Responders and non-responders were classified according to OMERACT-OARSI recommendations. Finally, an original cut-off method was applied to categorize patients and interpret individual variations in serum levels of Coll2-1.

**Results:**

Patients showed no difference in the sColl2-1 levels at baseline. When considering responders and non-responders from the ITT population, a significant difference was found for Coll2-1 at 3 months (*p* = 0.030) and 6 months (*p* = 0.038). A decrease in pain (VAS) and an improvement in function (LI) were recorded throughout the visits (*p* < 0.01). Considering an intra-batch cut-off of 21 %, CS decreased Coll2-1 serum levels between baseline and 1-month visit compared to the value of Coll2-1 before treatment (screening visit) which can be interpreted as a drastic reduction of the proportion of patients with an increase of Coll2-1 over 21 % (reduction from 13 to 3 %). It also consisted in a more important proportion of patients with a decrease in Coll2-1 (from 5 to 10 %).

**Conclusion:**

This study proposes a new approach for the analysis and the interpretation of the individual variation in biomarker levels and introduces the notion of metabolic responders.

**Trial registration:**

ID ISRCTN63795830. The trial was retrospectively registered on 2 October, 2015.

**Electronic supplementary material:**

The online version of this article (doi:10.1186/s12891-016-1268-4) contains supplementary material, which is available to authorized users.

## Background

Osteoarthritis (OA) is one of the most common forms of musculoskeletal disorders. It is one of the major cause of pain and disability in the adult population [1]. The challenge for the last decades has not only been to find a cure for OA but also to identify tools which could help the diagnosis and monitoring disease progression and efficacy of therapeutic interventions. Those tools need to be accurate for the monitoring of structural progression of the disease and sensitive enough to identify early event at the molecular level.

Biomarkers are among those possible tools. A biomarker is a characteristic that is objectively measured and evaluated as an indicator of normal biologic or pathogenic processes, or pharmacologic responses to a therapeutic intervention [2]. Biomarkers are not only essential for the understanding of pathological pathways but also for diagnosis, prognosis and follow up as previously described by Kraus et al. [3]. In addition, they could be valuable tool in the new era of personalized medicine.

Several biomarkers of bone, synovial membrane or cartilage metabolism have been proposed as potential tools for diagnosis, prognosis and monitoring of OA treatment [4]. Most of the developed biomarkers of cartilage degradation are epitopes located in type II collagen, one of the most specific molecules of the articular cartilage matrix [5, 6]. Collagen is degraded by enzymatic and non-enzymatic mechanisms in OA [7]. Included in the native form of the parental proteins, biomarkers are very often undetectable but when degradation processes occur, new epitopes become detectable and then can reflect the catabolic level present in the affected joint. Coll2-1 is a specific biomarker of cartilage degradation. Coll2-1 was indeed proven to be 2.5 times more elevated in OA patients than in healthy controls [8]. In addition it was shown to be a specific marker of cartilage degradation and to be correlated with the radiographic progression of OA [9].

Chondroitin sulfate (CS) is classified as a symptomatic slow-acting drug for the treatment of OA (SYSADOA) [10]. CS has demonstrated its effectiveness in improving pain and function in knee OA patients with a good safety profile [11–14]. Moreover CS was shown to be able to interfere with the pathophysiological process of OA and thereby to reduce the structural damage [12–17].

The aim of this study was to monitor the serum levels of Coll2-1 as primary endpoints in knee OA patients during a 6-month treatment with CS 800 mg/day and to measure the clinical efficacy of the treatment (i.e. pain and function) as secondary outcome.

## Methods

### Study design and patients

Unilateral symptomatic knee OA patients were recruited at the rheumatologist office of the Poal Institute of Rheumatology of Barcelona (Barcelona, Spain) from October 2012 to January 2014. This prospective, observational, post-authorization open label study (ID ISRCTN63795830) was reviewed and approved by the local health authorities and the institutional review board of IDIAP (Institut d’investigatiò en Atenciò Primaria Jordi Gol - Primary Care Research Institute) Jordi Gol i Gurina (Barcelona, Spain). Informed consent was obtained from all study participants. The study was performed according to the ethical principles of the Declaration of Helsinki and to Good Clinical Practice.

Patients responding to inclusion/exclusion criteria and considered by the physician as requiring a 6-month treatment of chondroitin sulfate (800 mg/day; Condrosan®, Bioiberica S.A., Spain) in accordance with the daily clinical practice were selected for the study. Serum levels of Coll2-1 (sColl2-1) and clinical evaluation (knee pain and functional incapacity) were monitored through five visits: selection visit (D-30), baseline visit (D0, initiation of treatment), 1-month visit, 3-month visit and 6-month visit.

The consumption of NSAIDs at anti-inflammatory doses was not allowed during the study. The use of acetaminophen as rescue medication was recorded throughout the study (inclusion and exclusion criteria are detailed below).

### Inclusion criteria

- Patients of both sexes and over 40 years of age diagnosed with unilateral symptomatic OA of the knee who met the criteria of the American College of Rheumatology (ACR) [18].
- Patients who were rated grade II or III on the Kellgren and Lawrence (K&L) radiological scale [19].
- Patients with symptomatic OA with a global mean pain in the knee >40 mm on a Visual Analogue Scale (VAS) for pain assessment.

### Exclusion criteria

- Women who were pregnant or breastfeeding.
- Patients with any form of decompensated or uncontrolled heart disease, diagnosed with renal or liver failure, severe infections, decompensated asthma, or a history of either alcoholism or another drug addiction and/or uncontrolled active psychiatric disorder.
- Patients who were grade I or IV on K&L radiological scale.
- Patients with bilateral symptomatic knee OA or symptomatic and developing OA in 3 or more joints, including the knee targeted in the study.
- Patients who have had a prosthesis replaced in the 12 months prior to inclusion.
- Concurrent joint rheumatisms (history and/or presence of signs at the time of selection) that could give rise to a misinterpretation of the evaluation of efficacy against pain or interfere in the evaluation, such as chondrocalcinosis, Paget’s disease of the limb which is ipsilateral in relation to the affected knee, rheumatoid arthritis, aseptic osteonecrosis, gout, septic arthritis, ochronosis, acromegaly, hemochromatosis, Wilson’s disease, osteochondromatosis, seronegative spondiloarthropathy, mixed conjunctival tissue disease, collagen vascular disease, psoriasis, inflammatory bowel disease.
- Participants who had a diagnosis of fibromyalgia (either by rheumatologist diagnosis, the 1990 American College of Rheumatology (ACR) criteria [20] or the Modified 2010 ACR (ACR) criteria [21].
- Patients who performed intense physical activity (daily or almost daily exercise practice).
- Patients with an osteotomy in the study knee.
- Arthroscopy in the previous 3 months.
- Patients with a contraindication for the use of CS.
- Patients who have used hyaluronic acid (intra-articular hyaluronic acid in the affected knee) during the 26 weeks prior to inclusion.
- Patients who have received intra-articular corticoid infiltrations in either their hips or knees in the 3 months prior to the intervention.
- Patients who had received oral corticoids in the 3 months prior to starting the study.
- Patients who had taken any of the drugs classified as SYSADOA in the three months prior to the baseline visit.
- Patients who had taken oral and/or topical NSAIDs (including COXIBs) at anti-inflammatory doses during the 30 days prior to the baseline visit.
- Patients who had used medicinal plants or homeopathic products and analgesic creams or gels during the week prior to inclusion.

### Populations

The safety population (SEP) corresponds to all the patients treated with at least one dose of the study medication.

The Intention-To-Treat (ITT) population includes all the patients who met inclusion and exclusion criteria, were treated with at least one dose of the study medication and had at least a baseline efficacy measurement, and at least one post-treatment measurement (1 month) for the primary efficacy variable, irrespective of their subsequent withdrawal from the study or protocol deviations.

The Protocol Population (PP) includes all the treated patients who met the following criteria:

- To meet the inclusion/exclusion criteria
- To be treated with at least one dose of the study medication
- To have consumed at least 75 % of the prescribed medication
- To have at least one baseline efficacy measurement and one post-treatment measurement for the primary efficacy variable
- To not present any major protocol deviation

Responders and non-responders were classified according to OMERACT-OARSI recommendations [22].

### Biomarker assay: sColl2-1

Coll2-1 has been determined in patients’ sera using ELISA kits according to manufacturers’ instructions (Artialis SA, Liège, Belgium).

### Evaluation of pain (VAS) and function (LI)

Mean pain during the last 24 h was measured on the Huskisson visual analog scale (VAS) [23] and function was evaluated through the algo-functional Lesquesne Index (LI) [24].

### Coll2-1 cut-off & physiological variability

Statistical analysis of data coming from clinical trials using Coll2-1 as biomarker of OA and comparing patients with or without treatment, or followed over time, commonly analyzed the results on a group basis using mean or median. This allows the identification of a significant modification in Coll2-1 concentration across the populations.

The intended use of Coll2-1 in clinical practice is its use as biomarker for the follow-up of patients with OA and the monitoring of the cartilage degradation. To this aim, each data should be analyzed on an individual basis and interpreted in term of modification of the cartilage metabolism for a single individual. To reach this point, we calculated the physiological variability of Coll2-1 concentration in serum which could not be related, for a single individual, between two time points, to a modification of the catabolism of the cartilage. The physiological variability of sColl2-1 was assessed on 15 asymptomatic subjects (NCT02348944) wherein Coll2-1 was measured at different sampling time. The group included ten non-menopausal women and five men (mean age: 31.6 +/−9.7 years). Intra-day and inter-day variability of sColl2-1 level was evaluated by collecting serum samples at four moments of the day on two different days:

- Sampling 1: early morning, after breakfast (~9 am)
- Sampling 2: at lunch time, before lunch (~12 am)
- Sampling 3: at lunch time, after lunch (~2 pm)
- Sampling 4: at end of a workday (~5 pm)

Day 1 of sampling at T0 was a midweek day (Tuesday) and Day 2 of sampling at T2 was right after the weekend (Monday) during the summer (August and September).

Instead of absolute values, the cut-off of physiological variability for sColl2-1 was determined in percentage change relative to baseline. This approach has been guided by several arguments. Indeed, Coll2-1 concentration showed a high inter-individual variability. Moreover, the use of percentage relative to baseline allows the comparison of different studies performed with different versions of the assay. The variability of sColl2-1 was determined with the following formula:

$$ \mathit{\mathsf{\varDelta sColl}}\mathit{\mathsf{2}}\mathit{\hbox{-}}\mathit{\mathsf{1}}\ \left(\%\right)=\left[\mathit{\mathsf{sColl}}\mathit{\mathsf{2}}\mathit{\hbox{-}}\mathit{\mathsf{1}}\ \left(\mathit{\mathsf{n}}\mathit{\mathsf{M}}\right)\mathit{\mathsf{Day}}\mathit{\mathsf{2}}\ \mathit{\hbox{-}}\ \mathit{\mathsf{sColl}}\mathit{\mathsf{2}}\mathit{\hbox{-}}\mathit{\mathsf{1}}\ \left(\mathit{\mathsf{n}}\mathit{\mathsf{M}}\right)\mathit{\mathsf{Day}}\mathit{\mathsf{1}}\right]/\left(\mathit{\mathsf{sColl}}\mathit{\mathsf{2}}\mathit{\hbox{-}}\mathit{\mathsf{1}}\ \left(\mathit{\mathsf{n}}\mathit{\mathsf{M}}\right)\mathit{\mathsf{Day}}\mathit{\mathsf{1}}\right) \times \mathit{\mathsf{1}\mathsf{00}} $$

The mean variability of Coll2-1 (ΔColl2-1) calculated for each subjects for each sampling time was 20.7 %. An absolute value of 21 % was chosen as a threshold above which the modification of Coll2-1 concentration is considered as significant and related to a modification of cartilage metabolism. This threshold designed categories of patients:

C: from −21 to +21 % = homeostasis; A: decrease in Coll2-1 catabolism > 21 %; E: increase in Coll2-1 catabolism.

### Statistical analysis

Two-group comparisons were performed with a Student T-test. Multiple comparisons were performed with an ANOVA followed by Bonferroni test. *P* value below 0.05 was considered statistically significant.

Two approaches were used to process missing data:

- “Last-Observation- Carried-Forward” method (LOCF), in which the value for the last observation was used to replace the missing piece of data. Using this method, in the case of patients withdrawn from the study, the last observation is treated as the final response of the patient, irrespective of the reason for withdrawal from the study or the value at the time of withdrawal.
- Available Case Analysis method (ACA), which exclusively employed complete data, ignoring incomplete study data.

## Results

### Population characteristics

Seventy two (72) unilateral symptomatic knee OA patients were selected for this study: 64 patients (nine men/55 women) were included in the analysis of the SEP, 61 (eight men/53 women) for the ITT population and 59 (eight men/51 women) for the PP population. Two (2) patients were excluded from the ITT population due to protocol deviations (both patients did not meet the wash-out period criteria for the allowed concomitant medication). The demographic characteristics (age, weight and BMI) of the study populations are detailed in Table 1.

**Table 1:** Demographic characteristics of the study populations

		Mean ± SD	Median	Min	Max
SEP	Age at selection visit (years)	59.00 ± 6.6	60.0	42	71
	Weight (kg)	70.7 ± 14.1	69.0	46.0	102.0
	BMI (kg/m^2^)	27.32 ± 4.71	26.85	18.29	38.05
ITT	Age at selection visit (years)	58.9 ± 6.6	60.0	42	71
	Weight (kg)	70.6 ± 14.0	70.0	46.0	102.0
	BMI (kg/m^2^)	27.39 ± 4.71	27.05	18.29	38.05
PP	Age at selection visit (years)	59.0 ± 6.7	60.0	42	71
	Weight (kg)	70.5 ± 14.11	70.0	46.0	102.0
	BMI (kg/m^2^)	27.33 ± 4.77	26.64	18.29	38.05

Patients from the ITT population were classified as responder or non-responder according to the OMERACT-OARSI recommendations [22]. Twenty three patients (23; 5 men/18 women) were considered as responders and 38 (three men/35 women) as non-responders. The demographic characteristics of the responders and non-responders are summarized in Table 2.

**Table 2:** Demographic characteristics of the responder and non-responder patients according to OMERACT-OARSI recommendations (22)

	Responders (*n* = 23)	Non-responders (*n* = 38)	Statistical significance
Age at selection visit (years)	58.00 ± 7.26	59.50 ± 6.31	0.421
Weight (kg)	69.0 ± 13.2	71.6 ± 14.5	0.492
BMI (kg/m^2^)	26.80 ± 3.4	27.8 ± 5.4	0.452

### Baseline values for sColl2-1 levels and pain

Patients from either ITT or PP populations showed no difference in the sColl2-1 levels at baseline. The levels in Coll2-1 serum in each population were respectively (mean +/− standard deviation) 1273.0+/−330.0 nM and 1273.0 +/− 332.0 nM. The same observation was made with responders and non-responders with different serum levels of biomarkers (data not shown) and pain (data not shown) at baseline.

### Primary endpoint: evolution of the serum levels of Coll2-1 (sColl2-1)

The comparison of sColl2-1 levels between screening (D-30) and baseline visit (D0; data not shown), period without treatment, revealed no variation in both ITT and PP populations, meaning that there was no variation without treatment.

When considering ITT and PP populations, no significant difference was reported throughout the follow up period. However, most biomarkers decreased after 1 month of treatment, though this reduction did not reach significance.

**Fig. 1:**
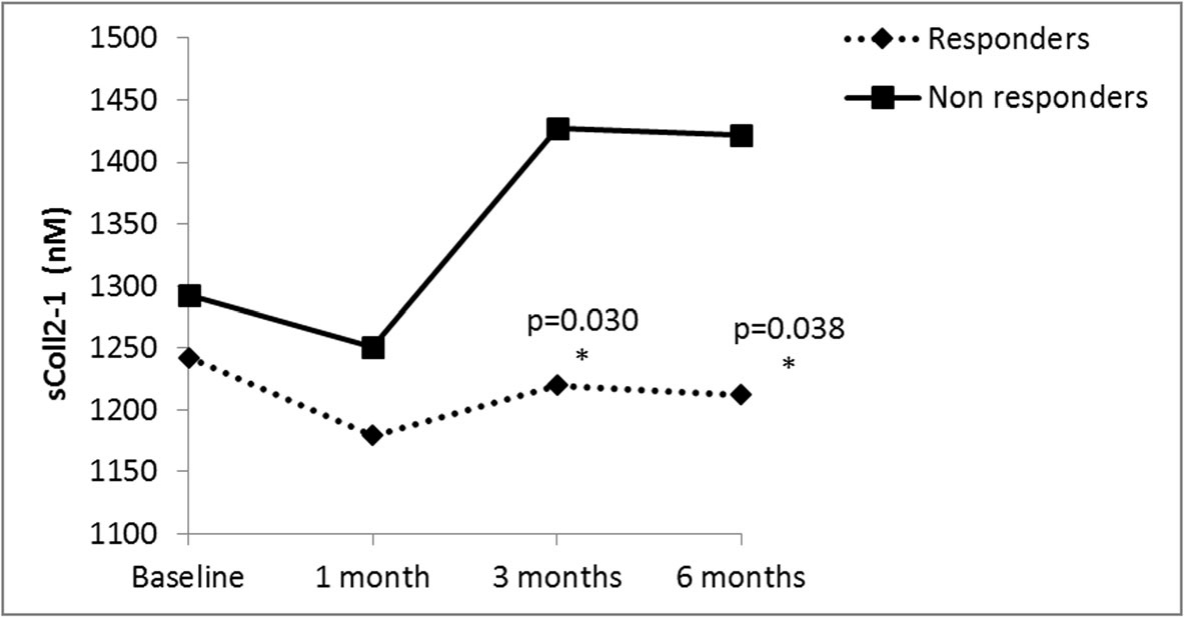
Serum levels of Coll2-1 in CS responder and non-responder knee OA patients

On the other hand, when considering responders and non-responders from the ITT population, a significant difference was found for Coll2-1 at 3 months (*p* = 0.030) and 6 months (*p* = 0.038) (ACA approach) (Fig. 1). A significant difference between both groups was also identified with the LOCF approach at 3 months (*p* = 0.0039).

### Secondary endpoints: evolution of pain (VAS) and function (LI)

A decrease in pain (VAS) and an improvement in function (LI) were recorded throughout the visits (Additional files 1 and 2). Using either LOCF or ACA approach, the decrease in pain was significant at 1, 3 and 6 months in ITT and PP populations when compared to baseline. The improvement of function was shown to be significant but to a lesser extent. Indeed significance was reached at the 6-month visit.

### Coll2-1 cut-off & physiological variability: results

Considering the categories described in Methods section *“Coll2-1 cut-off & physiological variability”*, the decrease of the Coll2-1 serum levels between baseline and the 1-month visit compared to the value of Coll2-1 before treatment (screening visit) can be interpreted as a drastic reduction of the proportion of patients considered as progressors in category E (reduction from 13 to 3 %; Table 3). Interestingly it also consisted in a more important proportion of patients with a decrease in Coll2-1 in category A (5 to 10 %). After 3 months of treatment, the percentage in category A (decrease in Coll2-1 catabolism >21 %) still increased from 10 to 20 %. However, the proportion of patient in category E, related to an increase in Coll2-1 catabolism, was also increased from 3 to 20 %. This was balanced by a reduction of patients included in the category C corresponding to physiological variability.

**Table 3:** Repartition of the study population in categories based on cut-off analysis

From screening to baseline			
(*n* = 63)	A	<−21 %	5 %
	C	−21 % to +21 %	83 %
	E	> + 21 %	13 %
From baseline to 1 month			
(*n* = 63)	A	<−21 %	10 %
	C	−21 % to +21 %	87 %
	E	> + 21 %	3 %
From baseline to 3 months			
(*n* = 60)	A	<−21 %	20 %
	C	−21 % to +21 %	60 %
	E	> + 21 %	20 %
From baseline to 6 months			
(*n* = 53)	A	<−21 %	11 %
	C	−21 % to +21 %	66 %
	E	> + 21 %	23 %

C = from −21 to +21 % (homeostasis); A = decrease in Coll2-1 catabolism >21 %; and E = Increase in Coll2-1 catabolism >21 %

## Discussion

Biomarkers are part of the challenge in OA research for the last decades. They have been proposed to monitor drug efficacy [3]. Their use in clinical trial is highly recommended [25] for their qualification. The other challenge in OA is to find a cure that could not only reduce symptoms (i.e. pain and function) but also improve structure.

CS efficacy was proven on OA symptoms in various clinical trials [11–14, 26]. The results of the present study are in the line of these previous trials. They are also in accordance with the SYSADOA definition [27]. Indeed the symptomatic effects are more pronounced after a several weeks of treatment. More recently CS was also shown to produce structural effect on the knee [17]. The structural benefit observed with MRI is partially the consequence of molecular events which occurred earlier in the joint tissues. Specific biomarkers could lead to an earlier detection of those effects, traducing a metabolic effect of the drug. The difference observed between responders and non-responders in Coll2-1 levels could illustrate this type of effect on the degradative event.

Biomarkers can be categorized according to the OA process targeted as markers of cartilage degradation/synthesis, bone remodeling, synovial tissue degradation/activity. The BIPEDS [3] classifies the major types of biomarkers according their clinical information into six categories corresponding to burden of disease, investigational, prognostic, efficacy of intervention, diagnostic biomarkers and safety biomarkers. In 2011, OARSI/FDA Osteoarthritis Biomarkers Working Group has classified biomarkers into four categories (exploration, demonstration, characterization and surrogacy levels) according to their current level of qualification for drug development [3, 28].

The qualification of a biomarker as a surrogate endpoint in clinical trial is paramount. The strategy is to correlate the variation of biomarker levels with clinical or imaging outcomes. However, a variation of soluble biomarker may also simply reflect a variation in tissue metabolism. Once could consider a reduction of cartilage catabolism as a therapeutic target. Therefore, based on the present results, one may wonder how to analyze and interpret the variation in biomarkers levels.

Coll2-1 was already shown to be reduced after a treatment, especially other SYSADOA [29, 30]. The sensitivity and accuracy of this biomarker have already been demonstrated. However, is it impossible to standardize the levels and variations of such biomarker in a disease as diversified as OA. Amidst the standard statistical analysis, the cutoff approach could be a solution. Indeed, the classification of patients in categories based not only on the assay performance but also on patient demographic characteristics for example could yield to a better analysis and to an illustration of the variation in the levels of biomarkers. Herein, we demonstrate that CS might modulate the ratio of “metabolic” responders. Furthermore we have observed that a ratio of patients showed an increase of serum Coll2-1 levels. It could be explained by contribution from other joints or by an increase of physical activity related to pain reduction. In addition to structural and functional responder, the notion of metabolic responder is introduced.

## Conclusions

Biomarkers are important tools for the monitoring of OA disease and of treatment. Herein, we demonstrate that CS may down-regulate cartilage catabolism in some but not all patients introducing the concept of “metabolic” responders. This study jeopardized the analysis and interpretation of the variation in biomarker levels. This analysis of biomarker variation should yield to an individual approach of patient follow up. Biomarkers should provide dynamic, metabolic and individual kinetic information.

## Additional files

Additional file 1: Evolution of function (LI). (DOCX 12 kb)
Additional file 2: Evolution of pain measured (VAS). (DOCX 12 kb)

## Abbreviations

ACA: Available case analysis
CS: Chondroitin sulfate
ITT: Intent-to-treat
K&L: Kellgren and Lawrence
LI: Lesquesne index
LOCF: Last-observation-carried-forward
MRI: Magnetic resonance imaging
OA: Osteoarthritis
PP: Protocol population
SEP: Safety population
SYSADOA: Symptomatic slow-acting drug for OA
VAS: Visual analog scale
