# Supplementary material for: Effect of chondroitin sulfate on soluble biomarkers of osteoarthritis: a method to analyze and interpret the results from an open-label trial in unilateral knee osteoarthritis patients
Source: BMC Musculoskelet Disord. 2016 Oct 6;17:416. doi: 10.1186/s12891-016-1268-4 (PMC5053075; doi:10.1186/s12891-016-1268-4)
Supplement: Additional file 1: — Evolution of function (LI). (DOCX 12 kb) [file 12891_2016_1268_MOESM1_ESM.docx]

**Effect of Chondroitin Sulfate on soluble Biomarkers of Osteoarthritis: a Method to Analyze and Interpret the Results from an Open-Label Trial in Unilateral Knee Osteoarthritis Patients**

Ingrid Möller^1^, Myriam Gharbi^2^, Helena Martinez Serrano^3^, Marta Herrero Barbero^3^, Josep Verges Milano^3^, Yves Henrotin^4^

**Additional Table 1**

| **Mean ± SD (n=61)** | **p Value** | **95% Confidence Interval (CI)** | |
| --- | --- | --- | --- |
|  |  | **Lower CI** | **Upper CI** |
| 64.02 ± 9.35 | --- | 61.62 | 66.41 |
| 57.92 ± 13.78 | 0.003* | 54.39 | 61.45 |
| 54.79 ± 18,27 | 0.002* | 50.11 | 59.47 |
| 50.56 ± 19.86 | 0.000* | 45.47 | 55.64 |
|  |  |  |  |
