# Supplementary material for: Effect of chondroitin sulfate on soluble biomarkers of osteoarthritis: a method to analyze and interpret the results from an open-label trial in unilateral knee osteoarthritis patients
Source: BMC Musculoskelet Disord. 2016 Oct 6;17:416. doi: 10.1186/s12891-016-1268-4 (PMC5053075; doi:10.1186/s12891-016-1268-4)
Supplement: Additional file 2: — Evolution of pain measured (VAS). (DOCX 12 kb) [file 12891_2016_1268_MOESM2_ESM.docx]

**Effect of Chondroitin Sulfate on soluble Biomarkers of Osteoarthritis: a Method to Analyze and Interpret the Results from an Open-Label Trial in Unilateral Knee Osteoarthritis Patients**

Ingrid Möller^1^, Myriam Gharbi^2^, Helena Martinez Serrano^3^, Marta Herrero Barbero^3^, Josep Verges Milano^3^, Yves Henrotin^4^

**Additional Table 2**

| **Mean ± SD (n=61)** | **p Value** | **95% Confidence Interval (CI)** | |
| --- | --- | --- | --- |
|  |  | **Lower CI** | **Upper CI** |
| 9.87 ± 4,47 | --- | 8.72 | 11.02 |
| 8.93 ± 4.56 | 0.113 | 7.77 | 10.1 |
| 8.51 ± 4.75 | 0.054 | 7.29 | 9.72 |
| 7.90 ± 4.52 | 0.007* | 6.74 | 9.06 |
